# Occurrence of *Cryptosporidium* spp. and *Cystoisospora belli* among adult patients with diarrhoea in Maputo, Mozambique

**DOI:** 10.1016/j.heliyon.2018.e00769

**Published:** 2018-09-07

**Authors:** Verónica Casmo, Marianne Lebbad, Salomão Maungate, Johan Lindh

**Affiliations:** a Instituto Nacional de Saúde, Maputo, Mozambique; b Department of Cell and Molecular Biology, Uppsala University, Uppsala, Sweden

**Keywords:** Microbiology

## Abstract

Infections with *Cryptosporidium* spp. and *Cystoisospora belli* are important causes of diarrhoea in HIV patients. Nevertheless, information concerning these two parasites is scarce in many African countries, including Mozambique. In this study occurrence of *Cryptosporidium* spp. and *C. belli* was investigated by microscopy of stool specimens from 108 adult diarrhoeal patients, most with a confirmed HIV diagnosis. The *Cryptosporidium* isolates were further characterized by molecular methods.

*Cryptosporidium* and *C. belli* oocysts were found in 8.3% (9/108), and 25.0% (27/108) of the study participants, respectively. Species identification was possible for all *Cryptosporidium* isolates with available DNA. The following *Cryptosporidium* species were detected (number of cases within parentheses): *C. parvum* (3)*, C. hominis* (3), *C. felis* (1)*,* and *C. hominis/C. parvum* (1). Subtyping targeting the gp60 gene revealed two *C. hominis* isolates with subtype IaA23R3, one *C. parvum* isolate with IIcA5G3d, and one with IIeA12G1.

In summary the occurrence of *C. hominis* and anthroponotic subtypes of *C. parvum* indicates that the main route of *Cryptosporidium* transmission in the present study population was human to human (direct or via food and water). The high prevalence of *C. belli* highlights the need for early diagnosis of this parasite, for which a treatment exists.

## 1 Introduction

The coccidian parasites *Cryptosporidium* and *Cystoisospora belli* (formerly *Isospora belli*) are important causes of diarrhoea among HIV-positive patients, especially in developing countries where these parasites are common. Most cases of human cryptosporidiosis are caused by *Cryptosporidium hominis* or *Cryptosporidium parvum,* and the latter species is responsible for most zoonotic infections in humans. However, some *C. parvum* subtypes have been found mainly in humans, which emphasises the need for molecular investigations to establish probable routes of infection for this species. Only anthroponotic transmission is considered for *C. belli*, because no animal reservoirs have been identified for this parasite.

In Mozambique, the role of *Cryptosporidium* in childhood diarrhoea has been intensively studied, but the occurrence of this parasite in adult HIV patients with diarrhoea is less investigated (Cerveja et al., 2017; Irisarri-Gutierrez et al., 2017; Kotloff et al., 2012; Nhampossa et al., 2015; Sow et al., 2016). Furthermore, data on *Cryptosporidium* species and subtypes in Mozambique are available for only a few isolates, which highlights the need for further research on this issue (Irisarri-Gutierrez et al., 2017; Sow et al., 2016). Few studies of the prevalence of intestinal parasites in Mozambique have involved *C. belli*, and local occurrence of this parasite has only been described twice; the first time in a case report concerning an AIDS patient with tuberculosis symptoms and watery diarrhoea and the second time in a HIV-negative patient originating from a health centre study (Cerveja et al., 2017; Clavero et al., 1999). Accordingly, we performed an observational study and investigated the incidence of *Cryptosporidium* species and subtypes as well as the occurrence of *C. belli* in adult patients with diarrhoea in Maputo, Mozambique.

## 2 Materials and methods

### 2.1 Patients

Patients at José Macamo General Hospital in Maputo were recruited between January 2011 and May 2013. In total, 108 patients (age ≥17 years) who had diarrhoea complaints (defined as ≥3 loose stools per day) and were admitted to the hospital were included in the study. The participants answered a questionnaire including items on sex, age, water sources, known HIV status, ongoing antiretroviral therapy (ART), and duration of diarrhoea.

### 2.2 Methods

All patients with unknown HIV status were offered HIV testing with HIV-1/2 (Abbott Laboratories, Tokyo, Japan) for screening and with Uni-Gold HIV (Trinity Biotech, Wicklow, Ireland) for confirmation. Stool samples were collected from all patients, one per patient. Smears were prepared directly from unpreserved stool specimens and then stained with modified Ziehl-Neelsen and investigated for the presence of coccidian oocysts (Henriksen and Pohlenz, 1981). A QIAamp DNA Stool Mini Kit (Qiagen, Hilden, Germany) was used according to the manufacturer's protocol to extract DNA from stool samples containing *Cryptosporidium* oocysts. *Cryptosporidium* spp. were evaluated by PCR targeting of the small subunit rRNA (SSU rRNA) gene followed by restriction fragment length polymorphism (RFLP) and sequencing (Xiao et al., 1999, 2001). *Cryptosporidium* subtypes were determined by PCR and subsequent sequencing of the 60-kDa glycoprotein (gp 60) gene (Alves et al., 2003, 2006). Samples with negative results in these two PCR assays were further investigated at the 70-kDa heat shock protein (hsp70) locus (Morgan et al., 2001). The sequences that were obtained were compared with sequences in the GenBank database (BLAST, https://blast.ncbi.nlm.nih.gov/Blast.cgi).

Representative sequences from this study have been deposited in GenBank (accession nos. KX579754–KX579757KX579754KX579755KX579756KX579757). Fisher's exact test was used for statistical analysis (Epi Info^TM^ CDC, Atlanta, GA, USA).

### 2.3 Ethical approval

Ethical approval for the investigation was granted by the National Committee for Bioethics for Health in Mozambique (12/CNBS/2009). An informed consent sheet was explained and given to all individuals who voluntarily agreed to participate in the study. Individuals who could not write were allowed to sign the consent form with an X. To ensure that the study objectives were understood by the participants, all information was provided in both Portuguese and a local language.

## 3 Results

**Fig. 1:**
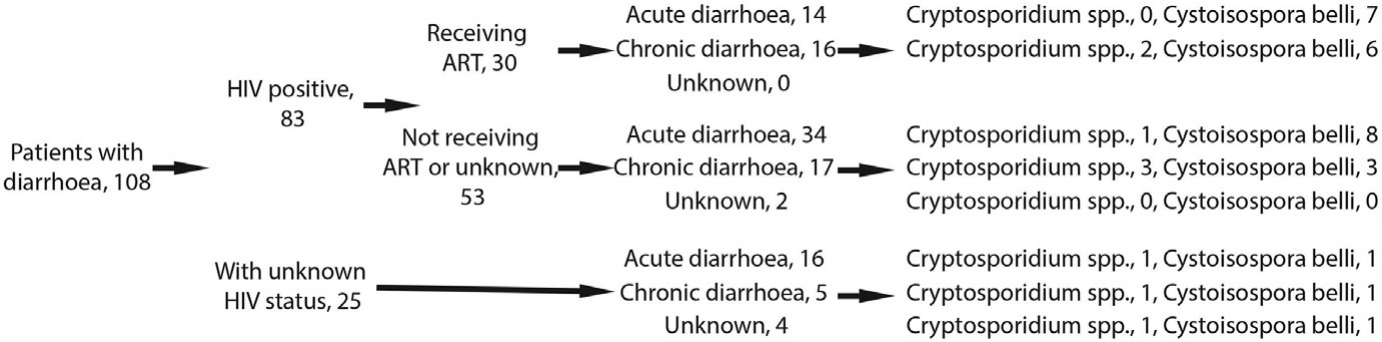
Flowchart demonstrating the status of 108 patients with diarrhoea.

**Fig. 2:**
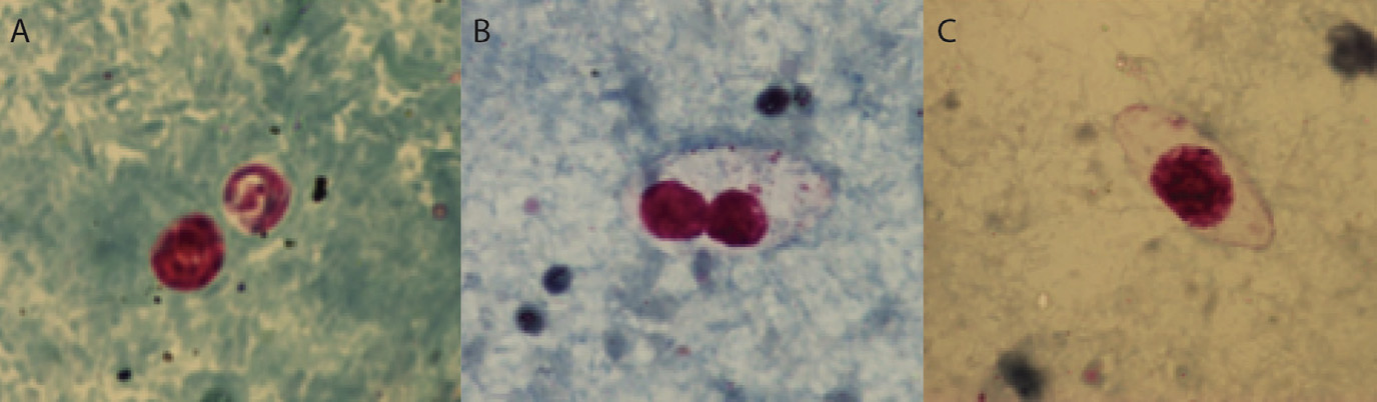
Coccidian oocysts stained with modified Ziehl-Neelsen technique (Henriksen and Pohlenz, 1981). Oocysts of *Cryptosporidium* spp. are round and measure 4–6 μm (A). Oocysts of *Cystoisospora belli* are long and oval shaped. The oocysts may vary a lot in size and a measure between 17–37 μm in length and 8–21 μm in width have been described (Jongwutiwes et al., 2007) B shows a mature oocyst with two sporocysts and C shows an immature oocyst with a single sporoblast.

In all, 108 patients (51 females, 57 males) were included in the study. The average age was 37.7 (17–73) years for females and 32.6 (19–60) years for males; data on age was missing for five patients (one female, four males). Eighty-three of the patients (42 females, 41 males) were HIV positive, whereas the HIV status of the remaining 25 patients (nine females, 16 males) had not been determined. Of the 83 patients with confirmed HIV infection, 30 (16 females, 14 males) were on ART, and 53 (26 females, 27 males) were not receiving treatment. Acute diarrhoea (<14 days) was reported by 64 of the patients (32 females, 32 males) and chronic diarrhoea (≥14 days) by 38 cases (19 females, 19 males). Duration of diarrhoea was unknown or inconclusive in six of the male cases (Fig. 1). Ninety participants reported having access to tap water, and 11 had well water; information regarding source of drinking water was missing for seven individuals, all males. Microscopy revealed *C. belli* oocysts in 25.0% (27/108) of the samples and *Cryptosporidium* oocysts in 8.3% (9/108), and two of these positive samples contained both parasites (Fig. 2). No oocysts of *Cyclospora cayetanensis* were detected. Identification of *Cryptosporidium* spp. was possible in all eight cases with available DNA, which revealed infections with the three species *C. parvum* (n = 3), *C. hominis* (n = 3)*,* and *Cryptosporidium felis* (n = 1), as well as one mixed infection with *C. hominis/C. parvum*. Subtyping at the gp60 level was successful for four isolates: two with *C. hominis* subtype IaA23R3, one with *C. parvum* IIeA12G1, and one with *C. parvum* IIcA5G3d. Information provided by the cryptosporidiosis patients is shown in Table 1, together with the results of molecular analyses. Likewise, information concerning the cystoisosporiasis patients is shown in Table 2. A weak but significant correlation was found between chronic diarrhoea and infection with *Cryptosporidium* spp. (p = 0.049), whereas no correlation was demonstrated between duration of diarrhoea and infection with *C. belli*. Moreover, no association was noted between *Cryptosporidium* or *C. belli* infections and the patients' sex, source of drinking water or ongoing antiretroviral therapy (all p values > 0.5).

**Table 1:** Information on *Cryptosporidium* species and subtypes in nine patients with cryptosporidiosis.

Patient ID	Sex	Age	HIV status	ART	Duration of diarrhoea	*Cryptosporidium* spp. rRNA/hsp70 reference acc. no.	gp60 subtype/reference acc. no	gp60 acc. no. in this study
Map04^a^	F	49	Positive	Yes	≥14 days	*C. hominis* rRNA GQ983350	IaA23R3 JQ798143	KX579754
Map22	M	U	U	U	U	*C. felis* hsp70 AF221538	-	
Map40	F	20	U	U	≥14 days	*C. parvum* rRNA JN812214	NA	
Map54	F	37	Positive	No	≥14 days	*C. hominis* rRNA GQ983350	IaA23R3 JQ798143	KX579755
Map60	F	33	Positive	Yes	≥14 days	*C. parvum* rRNA AF164102	IIeA12G1 AY382675^b^	KX579756
Map65	M	40	Positive	No	≥14 days	rRNA *C. hominis + C. parvum*^c^	IIcA5G3d AF440636	KX579757
Map70	F	33	Positive	No	≥14 days	*C. parvum* rRNA JN812214^d^	NA	
Map73^a^	M	29	Positive	No	<14 days	*C. hominis*, hsp70 XM_661662	NA	
Map108	M	43	U	U	<14 days	NP	NP	

NA: not amplified; NP: not performed (no DNA available). U: Unknown.

a Co-infected with *C. belli.*

b 99% identity (420/421 bp).

c Mixed species identified in the chromatogram.

d 99% identity (790/791 bp).

**Table 2:** Information on 27 patients with cystoisosporiasis.

Patient ID	Sex	Age	HIV status	ART	Duration of diarrhoea	No of *C. belli* oocysts/slide
Map02	M	40	Positive	Yes	≥14 days	11–50
Map04^a^	F	49	Positive	Yes	≥14 days	<5
Map10	F	60	U	U	≥14 days	U
Map12	M	27	U	U	<14 days	5–10
Map16	F	38	Positive	Yes	<14 days	<5
Map20	M	U	U	U	U	<5
Map27	F	61	Positive	No	<14 days	<5
Map32	M	46	Positive	Yes	<14 days	5–10
Map36	M	36	Positive	No	<14 days	<5
Map44	F	26	Positive	No	<14 days	<5
Map46	F	29	Positive	No	<14 days	11–50
Map49	F	35	Positive	Yes	<14 days	5–10
Map51	M	45	Positive	Yes	<14 days	11–50
Map53	M	28	Positive	No	≥14 days	11–50
Map55	M	32	Positive	Yes	<14 days	5–10
Map56	M	27	Positive	Yes	<14 days	>50
Map64	M	29	Positive	No	<14 days	<5
Map67	M	42	Positive	Yes	≥14 days	<5
Map68	M	27	Positive	No	<14 days	>50
Map69	F	29	Positive	Yes	≥14 days	<5
Map73^a^	M	29	Positive	No	<14 days	5–10
Map81	M	35	Positive	Yes	≥14 days	>50
Map87	M	34	Positive	No	≥14 days	<5
Map88	M	27	Positive	Yes	<14 days	11–50
Map90	M	30	Positive	No	≥14 days	<5
Map97	M	30	Positive	No	<14 days	<5
Map98	F	30	Positive	Yes	≥14 days	U

a Co-infected with *Cryptosporidium*. U: Unknown.

## 4 Discussion

This study conducted in Mozambique has specifically addressed the occurrence of *Cryptosporidium* and *C. belli* in adult diarrhoeic patients, most of whom had a confirmed diagnosis of HIV. Cryptosporidiosis in childhood diarrhoea has been studied in different areas of Mozambique, but adult diarrheic patients have only been investigated to a limited extent in this country (Cerveja et al., 2017; Irisarri-Gutierrez et al., 2017; Kotloff et al., 2012; Meurs et al., 2017; Nhampossa et al., 2015; Sow et al., 2016).

The prevalence of *Cryptosporidium* in Africa varies considerably, ranging from less than 1% in healthy children and adults to 72% in diarrheic patients (Squire and Ryan, 2017). We found *Cryptosporidium* oocysts in 8.3% of the participants in our study, which corroborates the results obtained in Chokwe in southern Mozambique in an investigation of *Cryptosporidium* and other intestinal protozoans in 99 HIV- and/or tuberculosis-infected individuals of various ages. Here cryptosporidiosis was diagnosed by nested PCR in 8% (8/99) of the evaluated patients, whereas no cases of cystoisosporiasis were reported, although only 15% of the participants had diarrhoea (Irisarri-Gutierrez et al., 2017). A similar prevalence in HIV-positive patients, 6% (8/201), was reported using a copro antigen test in a study focusing on HIV-positive and negative patients attending a health center in Maputo, while microscopy performed on all HIV-positive cases (371) revealed only one positive sample. In the same study one HIV-negative patient with *C. belli* oocysts was found (Cerveja et al., 2017). Another study from Beira, Mozambique, performed on 303 patients (general population, children and adults) reported a *Cryptosporidium* prevalence of 2% (6/302) using real time PCR while no *C. belli* oocysts were found in 201 samples investigated by microscopy (Meurs et al., 2017).

Subtyping of our isolates at the gp60 gene locus revealed the two *C. parvum* subtype families IIe and IIc, both of which are considered to be anthroponotic, because thus far they have been found mainly in humans. The subtype IIeA12G1 sequence was nearly identical to the reference sequence AY382675 (442/443 bases), but differed by around 18 bases in the post-repetitive part compared to several other sequences in GenBank dedicated to either IIe or IIm, and this observation underlines the need for harmonization of the nomenclature for these two subtype families. The IIeA12G1 subtype has been reported only twice, once in children in Malawi and once in HIV patients in Ethiopia (Adamu et al., 2010; Peng et al., 2003). In our study, RFLP and sequencing of the SSU rRNA gene revealed a patient with mixed *C. hominis/C. parvum* infection, but, despite repeated PCR/sequencing of the gp60 gene, only the *C. parvum* subtype, IIcA5G3d, was amplified. Many variants of IIcA5G3 (differentiated by lower-case suffixes) have been recognized. The subtype found in our study, IIcA5G3d, has been detected in children in South Africa and HIV-positive adults in Jamaica (Gatei et al., 2008; Leav et al., 2002). Sow and colleagues subtyped five *C. parvum* isolates from Mozambican children included in the Global Enteric Multicenter Study (GEMS) and according to their article the majority of the isolates were identified as IIcA5G3 (Sow et al., 2016). The two *C. hominis* isolates that could be subtyped in our investigation had subtype IaA23R3, which has previously been identified in the two African countries of Nigeria and São Tomé and Príncipe (Lobo et al., 2014; Maikai et al., 2012). Also, a recent study in Mozambique found two other *C. hominis* subtype families, Ib and Id, thus stressing the need for further genotyping efforts in this part of the world (Irisarri-Gutierrez et al., 2017). Two samples in our study, one with *C. hominis* and the other with *C. felis,* failed in both SSU rRNA and gp60 PCR but were positive in hsp70 PCR. A plausible explanation for these findings is that the hsp70 PCR we used amplifies a shorter fragment compared to the other two PCR assays we employed (325 vs. ∼900 bp) and hence is less sensitive to degraded DNA, and the DNA in our study was probably degraded after having been subjected to repeated freezing and thawing.

Considering that two of the four *C. parvum* isolates in our evaluation contained anthroponotic subtypes, and the other two could not be subtyped, the only support for potential zoonotic transmission was the infection with *C. felis*, a species that usually affects cats. Only a handful of studies conducted in Africa have described *C. felis* infections in humans (Squire and Ryan, 2017). Furthermore, only one investigation has considered the occurrence of *Cryptosporidium* in cats in Africa, and in that evaluation Samie and colleagues detected *Cryptosporidium* spp. in 32% of the cats in the Thohoyandou region in South Africa, an area close to Mozambique (Samie et al., 2013). However, it is possible that anthroponotic transmission of *C. felis* can occur, particularly in areas with a high incidence of cryptosporidiosis in HIV patients (Cama et al., 2006).

We found that 25.0% of the patients in our study were infected with *C. belli.* This rate is high compared to levels noted in most of the recent reports from Sub-Saharan Africa, indicating a prevalence of 0.7% in the general population of Burkina Faso and Mozambique (Cerveja et al., 2017; Sangare et al., 2015) and 3.5%–10% in HIV patients in Nigeria and Cameroon respectively (Olusegun et al., 2009; Vouking et al., 2014). Remarkably, in our study no differences in number of positive cases were seen between patients with or without ART provided, which would have been expected. We have no plausible explanation for this, but the higher rate we observed does concur with some investigations in India revealing cystoisosporiasis in 26–31% in patient groups similar to ours, HIV-positive patients with diarrhoea (Prasad et al., 2000; Vignesh et al., 2007). In another Indian study, also on HIV-positive patients with diarrhoea, all patients were provided ART, as well as trimethoprim/sulfamethoxazole (TMP/SMX) prophylaxis, despite that 22% were infected with *C. belli* (Mohanty et al., 2013). Thus it seems that although the incidence of *C. belli* might vary geographically, the differences in the number of diagnosed cases are mainly attributed to the immune and diarrhea status of the patients. Further on the sensitivity of the diagnostic methods used might contribute to differences in obtained prevalences (Bialek et al., 2002; Pacheco et al., 2013). In our investigation, where the entire Ziehl-Neelsen stained smears were observed by microscopy, the number of oocysts varied from one to >50 per sample, and thus cases with a low number of oocysts, which also varies considerably in size and appearance, could easily have been overlooked (Jongwutiwes et al., 2007) (Table 2, Fig. 2).

Cystoisosporiasis is a serious infection in immunosuppressed patients, but, in contrast to cryptosporidiosis, treatment, such as TMP/SMX, is available, and therefore it is very important to raise awareness about the occurrence of this parasite in HIV-infected individuals. TMP/SMX prophylaxis for *Pneumocystis jirovecii* pneumonia (pcp) is used in many settings for HIV-infected patients with a low CD4 count, and it might also be effective against the *C. belli* parasite (Anglaret et al., 1999). The national guidelines in Mozambique recommend that TMP/SMX be given as pcp prophylaxis to all HIV-positive patients (Guia de Tratamento Antiretroviral e Infecções Oportunistas no Adulto, 2013). Unfortunately, our study provided no information concerning this issue for the present patients.

In conclusion, an unexpectedly high occurrence of the diarrhoea-related coccidian parasite *C. belli* was found in this study. This observation, in combination with the presence of anthroponotic species/subtypes of *Cryptosporidium*, indicates that the human-to-human route represents the main pathway of transmission of coccidian intestinal parasites in the investigated population. Investigation of *C. belli* in HIV-positive subjects is advocated and should be included in routine diagnostic parasitology.

## Declarations

### Author contribution statement

Verónica Casmoa: Conceived and designed the experiments; Performed the experiments; Contributed reagents, materials, analysis tools or data.

Marianne Lebbad: Performed the experiments; Analyzed and interpreted the data; Wrote the paper.

Salomão Maungate: Performed the experiments.

Johan Lindh: Conceived and designed the experiments; Analyzed and interpreted the data; Contributed reagents, materials, analysis tools or data; Wrote the paper.

### Funding statement

This work was supported by the National Research Fund, Ministry of Technology and Science, Mozambique, and the Swedish International Development Cooperation Agency with funding provided via Programme 51140011 “Impact of Zoonotic Diseases on Public Health and Animal Production”.

### Competing interest statement

The authors declare no conflict of interest.

### Additional information

Data associated with this study has been deposited at GenBank under the accession number KX579754–KX579757KX579754KX579755KX579756KX579757.

## References

[bib1] Adamu H., Petros B., Hailu A., Petry F. (2010). Molecular characterization of *Cryptosporidium* isolates from humans in Ethiopia. Acta Trop..

[bib2] Alves M., Xiao L., Antunes F., Matos O. (2006). Distribution of Cryptosporidium subtypes in humans and domestic and wild ruminants in Portugal. Parasitol. Res..

[bib3] Alves M., Xiao L., Sulaiman I., Lal A.A., Matos O., Antunes F. (2003). Subenotype analysis of *Cryptosporidium* isolates from humans, cattle, and zoo ruminants in Portugal. J. Clin. Microbiol..

[bib4] Anglaret X., Chene G., Attia A., Toure S., Lafont S., Combe P., Manlan K., N'Dri-Yoman T., Salamon R. (1999). Early chemoprophylaxis with trimethoprim-sulphamethoxazole for HIV-1-infected adults in Abidjan, Cote d'Ivoire: a randomised trial. Cotrimo-CI Study Group. Lancet.

[bib5] Bialek R., Binder N., Dietz K., Knobloch J., Zelck U.E. (2002). Comparison of autofluorescence and iodine staining for detection of *Isospora belli* in feces. Am. J. Trop. Med. Hyg..

[bib6] Cama V., Gilman R.H., Vivar A., Ticona E., Ortega Y., Bern C., Xiao L. (2006). Mixed *Cryptosporidium* infections and HIV. Emerg. Infect. Dis..

[bib7] Cerveja B.Z., Tucuzu R.M., Madureira A.C., Nhacupe N., Langa I.A., Buene L., Funzamo C., Noormahomed E.V. (2017). Prevalence of intestinal parasites among HIV infected and HIV uninfected patients treated at the 1º de maio health centre in Maputo, Mozambique. EC Microbiol..

[bib8] Clavero A.O., Verdu M.E., Peman J., Dario R., Gobernado M. (1999). Human intestinal infection due to coccidia in Mozambique: two cases. Acta Trop..

[bib9] Gatei W., Barrett D., Lindo J.F., Eldemire-Shearer D., Cama V., Xiao L. (2008). Unique *Cryptosporidium* population in HIV-infected persons, Jamaica. Emerg. Infect. Dis..

[bib10] Guia de Tratamento Antiretroviral e Infecções Oportunistas no Adulto C. (2013). Adolescente e Grávida. http://docplayer.com.br/7447021-(Ficha-tecnica-titulo-guia-de-tratamento-antiretroviral-e-infeccoes-oportunistas-no-adulto-crianca-adolescente-e-gravida-2013.html.

[bib11] Henriksen S.A., Pohlenz J.F. (1981). Staining of cryptosporidia by a modified Ziehl-Neelsen technique. Acta Vet. Scand..

[bib12] Irisarri-Gutierrez M.J., Mingo M.H., de Lucio A., Gil H., Morales L., Segui R., Nacarapa E., Munoz-Antoli C., Bornay-Llinares F.J., Esteban J.G., Carmena D. (2017). Association between enteric protozoan parasites and gastrointestinal illness among HIV- and tuberculosis-infected individuals in the Chowke district, southern Mozambique. Acta Trop..

[bib13] Jongwutiwes S., Putaporntip C., Charoenkorn M., Iwasaki T., Endo T. (2007). Morphologic and molecular characterization of Isospora belli oocysts from patients in Thailand. Am. J. Trop. Med. Hyg..

[bib14] Kotloff K.L., Blackwelder W.C., Nasrin D., Nataro J.P., Farag T.H., van Eijk A., Adegbola R.A., Alonso P.L., Breiman R.F., Faruque A.S., Saha D., Sow S.O., Sur D., Zaidi A.K., Biswas K., Panchalingam S., Clemens J.D., Cohen D., Glass R.I., Mintz E.D., Sommerfelt H., Levine M.M. (2012). The Global Enteric Multicenter Study (GEMS) of diarrheal disease in infants and young children in developing countries: epidemiologic and clinical methods of the case/control study. Clin. Infect. Dis..

[bib15] Leav B.A., Mackay M.R., Anyanwu A., RM O.C., Cevallos A.M., Kindra G., Rollins N.C., Bennish M.L., Nelson R.G., Ward H.D. (2002). Analysis of sequence diversity at the highly polymorphic Cpgp40/15 locus among *Cryptosporidium* isolates from human immunodeficiency virus-infected children in South Africa. Infect. Immun..

[bib16] Lobo M.L., Augusto J., Antunes F., Ceita J., Xiao L., Codices V., Matos O. (2014). *Cryptosporidium* spp., *Giardia duodenalis, Enterocytozoon bieneusi* and other intestinal parasites in young children in Lobata province, Democratic Republic of Sao Tome and Principe. PLoS One.

[bib17] Maikai B.V., Umoh J.U., Lawal I.A., Kudi A.C., Ejembi C.L., Xiao L. (2012). Molecular characterizations of *Cryptosporidium, Giardia*, and *Enterocytozoon* in humans in Kaduna state, Nigeria. Exp. Parasitol..

[bib18] Meurs L., Polderman A.M., Vinkeles Melchers N.V., Brienen E.A., Verweij J.J., Groosjohan B., Mendes F., Mechendura M., Hepp D.H., Langenberg M.C., Edelenbosch R., Polman K., van Lieshout L. (2017). Diagnosing Polyparasitism in a High-Prevalence Setting in Beira, Mozambique: detection of Intestinal Parasites in Fecal Samples by Microscopy and Real-Time PCR. PLoS Negl Trop Dis.

[bib19] Mohanty I., Panda P., Sahu S., Dash M., Narasimham M.V., Padhi S., Parida B. (2013). Prevalence of isosporiasis in relation to CD4 cell counts among HIV-infected patients with diarrhea in Odisha, India. Adv. Biomed. Res..

[bib20] Morgan U.M., Monis P.T., Xiao L., Limor J., Sulaiman I., Raidal S., O'Donoghue P., Gasser R., Murray A., Fayer R., Blagburn B.L., Lal A.A., Thompson R.C. (2001). Molecular and phylogenetic characterisation of *Cryptosporidium* from birds. Int. J. Parasitol..

[bib21] Nhampossa T., Mandomando I., Acacio S., Quinto L., Vubil D., Ruiz J., Nhalungo D., Sacoor C., Nhabanga A., Nhacolo A., Aide P., Machevo S., Sigauque B., Nhama A., Kotloff K., Farag T., Nasrin D., Bassat Q., Macete E., Levine M.M., Alonso P. (2015). Diarrheal Disease in Rural Mozambique: burden, Risk Factors and Etiology of Diarrheal Disease among Children Aged 0-59 Months Seeking Care at Health Facilities. PLoS One.

[bib22] Olusegun A.F., Okaka C.E., Luiz Dantas Machado R. (2009). Isosporiasis in HIV/AIDS patients in Edo state, Nigeria. Malays. J. Med. Sci..

[bib23] Pacheco F.T., Silva R.K., Martins A.S., Oliveira R.R., Alcantara-Neves N.M., Silva M.P., Soares N.M., Teixeira M.C. (2013). Differences in the detection of *Cryptosporidium* and *Isospora (Cystoisospora*) oocysts according to the fecal concentration or staining method used in a clinical laboratory. J. Parasitol..

[bib24] Peng M.M., Meshnick S.R., Cunliffe N.A., Thindwa B.D., Hart C.A., Broadhead R.L., Xiao L. (2003). Molecular epidemiology of cryptosporidiosis in children in Malawi. J. Eukaryot. Microbiol..

[bib25] Prasad K.N., Nag V.L., Dhole T.N., Ayyagari A. (2000). Identification of enteric pathogens in HIV-positive patients with diarrhoea in northern India. J. Health Popul. Nutr..

[bib26] Samie E.A., Tsipa M.A., Bessong P. (2013). The epidemiology of *Cryptosporidium* in cats and dogs in the Thohoyandou region, South Africa. Afr. J. Microbiol. Res..

[bib27] Sangare I., Bamba S., Cisse M., Zida A., Bamogo R., Sirima C., Yameogo B.K., Sanou R., Drabo F., Dabire R.K., Guiguemde R.T. (2015). Prevalence of intestinal opportunistic parasites infections in the University hospital of Bobo-Dioulasso, Burkina Faso. Infect Dis. Poverty.

[bib28] Sow S.O., Muhsen K., Nasrin D., Blackwelder W.C., Wu Y., Farag T.H., Panchalingam S., Sur D., Zaidi A.K., Faruque A.S., Saha D., Adegbola R., Alonso P.L., Breiman R.F., Bassat Q., Tamboura B., Sanogo D., Onwuchekwa U., Manna B., Ramamurthy T., Kanungo S., Ahmed S., Qureshi S., Quadri F., Hossain A., Das S.K., Antonio M., Hossain M.J., Mandomando I., Nhampossa T., Acacio S., Omore R., Oundo J.O., Ochieng J.B., Mintz E.D., O'Reilly C.E., Berkeley L.Y., Livio S., Tennant S.M., Sommerfelt H., Nataro J.P., Ziv-Baran T., Robins-Browne R.M., Mishcherkin V., Zhang J., Liu J., Houpt E.R., Kotloff K.L., Levine M.M. (2016). The Burden of *Cryptosporidium* Diarrheal Disease among Children < 24 Months of Age in Moderate/High Mortality Regions of Sub-Saharan Africa and South Asia, Utilizing Data from the Global Enteric Multicenter Study (GEMS). PLoS Negl. Trop. Dis..

[bib29] Squire S.A., Ryan U. (2017). *Cryptosporidium* and *Giardia* in Africa: current and future challenges. Parasites Vectors.

[bib30] Vignesh R., Balakrishnan P., Shankar E.M., Murugavel K.G., Hanas S., Cecelia A.J., Thyagarajan S.P., Solomon S., Kumarasamy N. (2007). High proportion of isosporiasis among HIV-infected patients with diarrhea in southern India. Am. J. Trop. Med. Hyg..

[bib31] Vouking M.Z., Enoka P., Tamo C.V., Tadenfok C.N. (2014). Prevalence of intestinal parasites among HIV patients at the Yaounde Central Hospital, Cameroon. Pan Afr. Med. J..

[bib32] Xiao L., Bern C., Limor J., Sulaiman I., Roberts J., Checkley W., Cabrera L., Gilman R.H., Lal A.A. (2001). Identification of 5 types of *Cryptosporidium* parasites in children in Lima, Peru. J. Infect. Dis..

[bib33] Xiao L., Morgan U.M., Limor J., Escalante A., Arrowood M., Shulaw W., Thompson R.C., Fayer R., Lal A.A. (1999). Genetic diversity within Cryptosporidium parvum and related Cryptosporidium species. Appl. Environ. Microbiol..

